# Editorial: Neural Modulation of Conscious Perception: Emerging Approaches From Basic Research to Clinical Translation

**DOI:** 10.3389/fpsyg.2021.779798

**Published:** 2021-10-22

**Authors:** Luca Ronconi, Marcello Maniglia, Luca Battaglini, David Melcher

**Affiliations:** ^1^School of Psychology, Vita-Salute San Raffaele University, Milan, Italy; ^2^Division of Neuroscience, Scientific Institute San Raffaele Hospital, Milan, Italy; ^3^Department of Psychology, University of California, Riverside, Riverside, CA, United States; ^4^Department of Neurobiology, University of Alabama at Birmingham, Birmingham, AL, United States; ^5^Department of General Psychology, University of Padua, Padua, Italy; ^6^Neuro Vis. U.S. Laboratory, University of Padua, Padua, Italy; ^7^Department of Physics and Astronomy “Galileo Galilei,” University of Padua, Padua, Italy; ^8^Department of Psychology and Cognitive Science, University of Trento, Trento, Italy; ^9^Psychology Program, Division of Science, New York University Abu Dhabi, Abu Dhabi, United Arab Emirates

**Keywords:** consciousness, visual awareness, visual perception, non-invasive brain stimulation, active perception

Unveiling the neural correlates of conscious perception, and consciousness in general, is a multifaceted problem that needs to take into account, among other factors, how we encode and update stimulus information in a dynamic environment (Dehaene and Changeux, 2011). The present Research Topic starts from this perspective and focuses on studies employing non-invasive brain stimulation (NIBS) techniques to clarify the neural mechanisms involved in conscious perception and the application of this knowledge in clinical settings.

Concerning the latter, NIBS may provide new rehabilitative approaches in patients with disorders of consciousness (DOC) (Angelakis et al., 2014), and this Research Topic provides two such contributions. In their pilot study, Zhang, Liu, Li, Li et al. used transcranial direct current stimulation (tDCS) to reduce psychomotor inhibition state (PIS), a major problem for patients with DOC recovering from traumatic brain injury. Results reveal that after 40 sessions of anodal-tDCS over prefrontal areas, 10 patients showed improvement in the JFK Coma Recovery Scale-Revised and in the Apathy Evaluation Scale, suggesting an improvement in PIS. In another study from the same group (Zhang, Liu, Li, Duan et al.) with a larger sample of patients with DOC, the authors showed that a multi-target tDCS protocol that stimulates temporal and prefrontal cortices of both hemispheres in subsequent sessions can ameliorate patients' outcomes as assessed by common clinical scales. Moreover, in both studies non-linear indices of electroencephalographical (EEG) activity showed evidence for reorganization in both the affected and unaffected hemispheres.

While clinical applications of NIBS are showing promise toward new interventions for DOC, other contributions to the Research Topic articles demonstrate new technological, methodological and theoretical developments in exploring how conscious perception emerges from brain activity. Dalong et al. used cathodal tDCS (c-tDCS) and neuroimaging to investigate the role of the right temporo-parietal junction (TPJ) in a task involving self-motion in a rotational vestibular chair. C-tDCS over the right TPJ significantly reduced position and duration estimation in the contralateral side of stimulation. Resting state fMRI further revealed reduction of functional connectivity between TPJ and spatial perception-related cortical regions. These results suggest that the TPJ may encode vestibular-guided movement in a form that preserves the relationship between traveled distance, motion velocity, and motion duration.

A key aspect of visual awareness is how processing of different features, involving specific brain areas, can yield unified percepts. Donato et al. provide a critical review of the influential theory positing the anatomical segregation of the visual system into a dorsal stream, responsible for the processing of motion and spatial relationships between visual elements, and a ventral stream that computes form and orientation (Ungerleider and Mishkin, 1982; Ungerleider and Haxby, 1994). Donato et al. discuss recent studies that paint a more complex relationship between form and motion processing, which requires recurrent connectivity between dorsal and ventral visual regions to integrate spatial and temporal characteristics of objects in motion.

There is growing interest in the role of neural oscillations in conscious perception, in particular how rhythmic brain activity at multiple temporal scales may index neural excitability states and support the communication between different cortical regions (Fries, 2015; Ronconi et al., 2016; Bonnefond et al., 2017). The use of transcranial alternating current stimulation (tACS) as a selective way to modulate task-relevant neural oscillations is a more recent approach to uncovering the role of specific oscillatory activity (Cabral-Calderin and Wilke, 2020; Kasten and Herrmann, 2020). In this Research Topic Battaglini et al. tackle the long-standing question of how the visual stream is parsed into discrete or quasi-discrete units such that, for example, two successive visual stimuli are interpreted as simultaneous if separated by short time interval (20–50 ms; Stroud, 1967; VanRullen, 2016; Ronconi et al., 2017). The authors showed that occipital tACS in the alpha range (but not in the beta range) over lateral occipital areas including V5/MT induces participants to integrate two brief stimuli into a unique percept more often, suggesting a reduction of the temporal resolution of perception and a modulation of the extension of its constituent units. These findings are in line with what emerged from the review article of Ghiani et al., that represents an attempt to delineate a causal link between specific brain rhythms in particular cortical locations and the active construction of our conscious perception, including spatial, temporal and feature binding. Even though strong conclusions are not possible given the limited number of studies so far, initial findings suggest that spatial binding may be modulated using tACS in the theta and beta band in parietal areas, while (spatio)-temporal binding has been successfully modulated by stimulating parieto-occipital areas at gamma and alpha rhythms.

Limiting the investigation of perceptual awareness to how we process incoming stimuli might neglect the central role of action and our interactions with the environment, which demand a continuous dynamic adjustment and re-evaluation of our temporary representations of sensory input (Hecht et al., 2001). Using single-pulse transcranial magnetic stimulation (TMS), Hobot et al. investigated whether the activation of the motor system could play a role in visual awareness. TMS to M1, as compared to TMS on a control site, resulted in higher average awareness ratings, with the amplitude of the motor-evoked potentials (MEPs) that was correlated with visual awareness ratings. They conclude that externally induced activity in motor areas served as additional information for the evidence-accumulation in visual awareness.

In cases of ambiguous natural scenes, such as when dealing with noisy, occluded or complex visual input, the interpretation of elements embedded in natural scenes can even lead to perceptions of non-existent entities, a phenomenon called pareidolia or projection (Liu et al., 2014). These phenomena—beyond their long-standing clinical use—represent an interesting point of view to study our tendency to form Gestalts and make sense of patterns in the visual field (Gregory, 2000). The study by Bartel et al. deals with this fascinating topic; using ambiguous inkblots from the Rorschach test in a randomized double-blind design, they found that a-tDCS over the left prefrontal cortex (PFC) increased the number of meaningful patterns recognized in the inkblots as compared to the control stimulation, but did not influence performance on common tasks of visual insight. The study suggests a causal role of the left PFC in the top-down matching between perceptual and semantic representation.

Taken together, these studies provide an overview and contribution to the study of perhaps the most fundamental mystery in cognitive science: the brain mechanisms responsible for conscious perception. These studies champion promising experimental techniques, paradigms and theories that promise to further push the envelope in this field.

## Author Contributions

All authors contributed to the conceptualization and writing of this Editorial article.

## Conflict of Interest

The authors declare that the research was conducted in the absence of any commercial or financial relationships that could be construed as a potential conflict of interest.

## Publisher's Note

All claims expressed in this article are solely those of the authors and do not necessarily represent those of their affiliated organizations, or those of the publisher, the editors and the reviewers. Any product that may be evaluated in this article, or claim that may be made by its manufacturer, is not guaranteed or endorsed by the publisher.
